# Suicide

**Published:** 1908-07-04

**Authors:** 


### SUICIDE.

Suicide is one of the sociological phenomena for which hitherto no adequate explanation has been offered. That it occurs in practically every civilised state must be admitted, and, so far as England and Wales are concerned, it is argued that there is a considerable increase. There is also an increase in the number of insane quite disproportionate to the increase of population generally. It might be hastily assumed that there is some significant connection between these two phenomena, but there does not appear to be any ground for such an assumption. For example, if certain countries of Europe be taken and arranged according to the percentage of suicides, and then according to that of the insane, it will be found that in the first department Scotland is eighth, in the other second; while Saxony is precisely the reverse. Moreover, there are considerably more insane women than men, though the rate of suicide is much higher among men. Again, it might be supposed that a month like November, which in England seems to touch the nadir of discomfort, would be particularly rich in suicides; but such is not the case. The greatest number of suicides take place in the months of June and July. Another interesting consideration is the state of the individual, whether he be married or single, with children or childless. The aged bachelor is first, the married man without children second, and lastly, with a very small percentage, the man with a family. A further point worth investigation is the religion professed by various countries, particularly the German provinces, and the possible connection with suicide. It was found, though the statistics are not absolutely uniform, that the rate of suicide is higher in Protestant than in Roman Catholic districts.

It is scarcely possible as yet to base any really adequate theory upon the facts given above, though it may not be entirely superfluous to point to certain conclusions which require further evidence. Going back to the animal world, it seems impossible to find any really authenticated case of suicide. There are, of course, numerous floating examples which are the subject of club-room conversations. The snake that stings itself when surrounded by fire, the dog that refuses to eat on the death of its master, the horse of like principles, the mutilated wasp—all these are quoted without hesitation, though their existence in fact is doubtful. Even if there were cases in the animal world, we should have to be chary of recognising them, since suicide must *ipso facto* be bound up with self-consciousness. From the fact that elderly bachelors show such a high percentage, we might be inclined to infer, not that marriage is a blessed state, but that, failing all else, it gives a man an interest in life which he otherwise has not. The significance of the month June is very difficult to explain. We might assign as a reason the rise in temperature and increase in work pressure, though this seems to contradict the theory that absence of interest is a concomitant or antecedent of suicide. The religious question would probably resolve itself into a question of greater or lesser social integration, a problem of the same kind as that of marriage. To all this the medical man can at once add cases of suicide which may be the result of specific diseases, as also will the alienist cite instances of madness with suicidal tendencies. There is great need of careful investigation. It might, for example, be discovered that suicide is connected with deficient nerve-supply, an explanation used to explain the eccentricities of waltzing mice. Probably the discovery of the actual condition of brain in those who commit suicide is very far off, but this should not prevent the careful accumulation of statistics, which may give the clue for the beginning of experiment.

